# ATAC-seq on biobanked specimens defines a unique chromatin accessibility structure in naïve SLE B cells

**DOI:** 10.1038/srep27030

**Published:** 2016-06-01

**Authors:** Christopher D. Scharer, Emily L. Blalock, Benjamin G. Barwick, Robert R. Haines, Chungwen Wei, Ignacio Sanz, Jeremy M. Boss

**Affiliations:** 1 Department of Microbiology and Immunology, Emory University School of Medicine, Atlanta, GA 30322, USA; 2 Department of Rheumatology, Emory University School of Medicine, Atlanta, GA 30322, USA

## Abstract

Biobanking is a widespread practice for storing biological samples for future studies ranging from genotyping to RNA analysis. However, methods that probe the status of the epigenome are lacking. Here, the framework for applying the Assay for Transposase Accessible Sequencing (ATAC-seq) to biobanked specimens is described and was used to examine the accessibility landscape of naïve B cells from Systemic Lupus Erythematosus (SLE) patients undergoing disease flares. An SLE specific chromatin accessibility signature was identified. Changes in accessibility occurred at loci surrounding genes involved in B cell activation and contained motifs for transcription factors that regulate B cell activation and differentiation. These data provide evidence for an altered epigenetic programming in SLE B cells and identify loci and transcription factor networks that potentially impact disease. The ability to determine the chromatin accessibility landscape and identify *cis*-regulatory elements has broad application to studies using biorepositories and offers significant advantages to improve the molecular information obtained from biobanked samples.

Biorepositories are a growing and important source of biological specimens that allow researchers access to large cohorts of samples that would otherwise be unobtainable. Protocols for extraction and molecular phenotyping of DNA and RNA from biobanked specimens have been developed^1^. However, methods examining the epigenetic state of biobanked cells are lacking. Epigenetic information has the potential to reveal details about the molecular programming of cells, including the location and status of *cis*-regulatory elements. For example, the mapping of intergenic regulatory elements combined with traditional GWAS studies could improve the functional understanding of non-coding polymorphisms. However, it is not known whether the biobanking process preserves chromatin structure, thereby facilitating or inhibiting such analyses.

The Assay for Transposase Accessible Chromatin (ATAC-seq) utilizes a sequencing adapter-coupled Tn5 transposase to simultaneously tag and fragment native chromatin, thereby generating a high-resolution map of accessible loci from cells^2^. ATAC-seq is highly efficient, requires fewer cells than other epigenetic profiling assays, such as ChIP-seq, and can be used as a readout to predict epigenetic states. Here, the ATAC-seq assay was applied to both biobanked and freshly processed specimens and an indistinguishable chromatin accessibility pattern was observed. To validate the use of ATAC-seq on clinically biobanked specimens, the chromatin accessibility landscape was determined for naïve B cells isolated from an existing biorepository of Systemic Lupus Erythematosus (SLE) samples. Differentially accessible loci suggested a unique accessibility signature of SLE B cells and highlight transcription factor networks and loci that may contribute to disease.

## Results

### B cell complexity is preserved through biobanking

**Figure 1:**
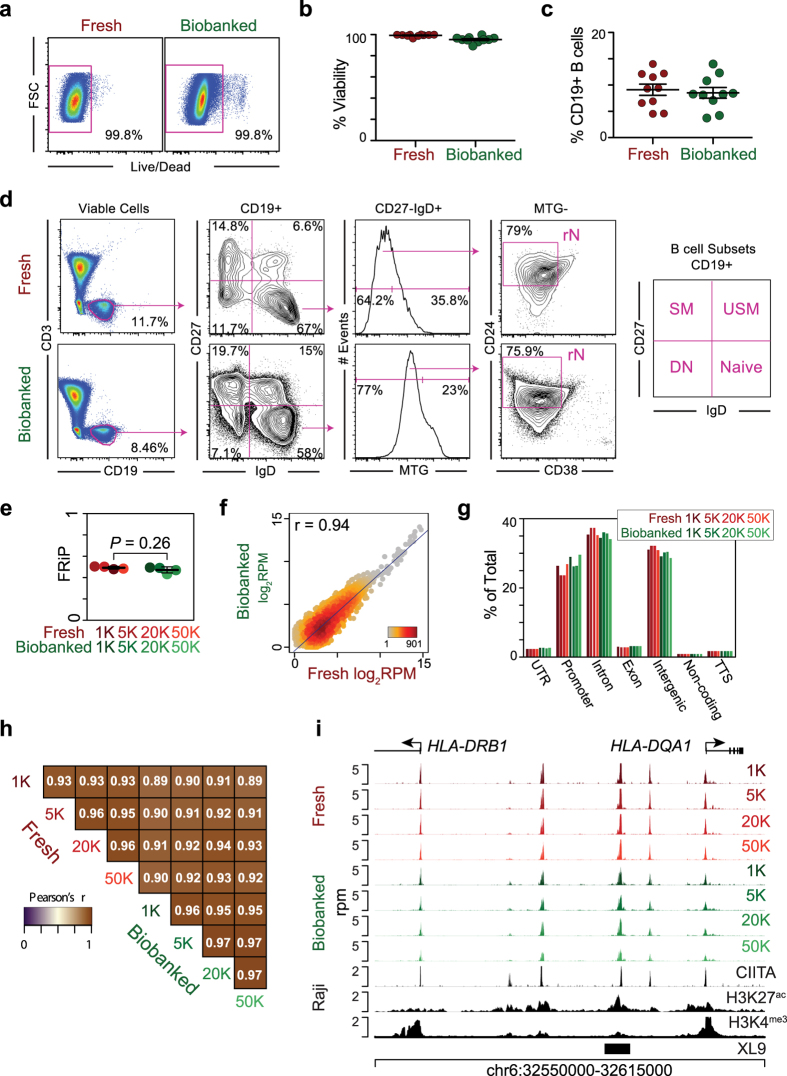
Biobanked samples display indistinguishable chromatin accessibility profiles from freshly processed samples. (**a**) Representative flow cytometry plots of cellular viability for fresh and biobanked specimens. Samples are gated on FSC and SSC prior to viability analysis. (**b**) Dot plot of the percentage viability for ten fresh and ten biobanked specimens. (**c**) Dot plot showing the percentage of CD19^+^ B cells for ten fresh and ten biobanked samples. (**d**) Flow cytometry analysis showing the phenotype of B cell subsets from a representative fresh and biobanked sample and the gating strategy to isolate naïve B cells (rN). The distinct CD19^+^ B cell subsets are indicated on the right. SM, switched memory; USM, unswitched memory; DN, double negative. (**e**) The fraction of reads in peaks (FRiP) metric for each sample is plotted. Statistical significance was tested by Student’s *t*-test. (**f**) Density scatter plot of the ATAC-seq reads in 76,591 combined accessible peaks from fresh and biobanked samples. Pearson’s correlation coefficient r value is indicated along with the scatter plot density. rpm, reads per million. (**g**) Barplot representing the percentage of peaks in each sample overlapping distinct genomic features. TTS, transcription termination site. UTR, 5′ and 3′ UTRs. (**h**) Heatmap of the Pearson’s correlation r values for all sample comparisons. The r value for each comparison is indicated. (**i**) Genome plot of the MHCII locus showing the profile for each ATAC-seq sample. Genomic profiles for CIITA, H3K27^ac^, and H3K4^me3^ from Raji B lymphoblastoid cells^6^ and the position of the XL9 insulator^5^ are plotted. The genomic coordinates, positions of genes, direction of transcription, and exon locations are annotated.

To facilitate the storage and sharing of clinical samples within and between institutions of the Autoimmunity Centers of Excellence, a robust PBMC biobanking protocol was established. Following thawing and preparation for FACS sorting, a near identical cellular viability was observed for biobanked specimens compared to freshly isolated samples (Fig. 1a,b). Additionally, the biobanking process maintained B cell complexity as determined by the frequency of peripheral CD19^+^ B cells (Fig. 1c) and distinct B cell subsets (Fig. 1d and Supplementary Fig. S1a,b). These data demonstrated that biobanked cells are viable and display surface markers similar to freshly processed cells.

### Chromatin structure is preserved through biobanking

To determine if the biobanking process preserved chromatin structure, thus facilitating the determination of epigenetic states, a study applying the ATAC-seq assay to fresh and biobanked human B cells was performed. PBMCs from a single healthy donor were split in half and processed fresh or biobanked for one week. Next, CD19^+^ naive B cells (IgD^+^ CD19^+^ MTG^−^CD27^−^CD38^+^ CD24^+^) were FACS isolated from both fresh and biobanked samples. To determine if there were cell input limitations associated with biobanking, 1,000, 5,000, 20,000, and 50,000 cells were isolated from each sample and ATAC-seq was performed. Accessible peaks of enrichment were determined and the fraction of reads in peaks (FRiP) was calculated. The FRiP metric can be used to assess background in enrichment assays^3^. No difference between the biobanked and fresh samples was observed (Fig. 1e), suggesting identical signal to noise ratios and that tagmentation – the process of tagging and fragmenting accessible chromatin during ATAC-seq – occurred primarily at focal accessible regions. The correlation of accessibility levels in peaks identified across all samples indicated an indistinguishable accessible chromatin landscape (Fig. 1f). Furthermore, the overlap of peaks across a wide range of genomic annotations indicated that either biobanking or reducing the starting cell number did not bias the discovery of certain genomic features (Fig. 1g). Also, the distribution of accessible intergenic, intronic, and promoter regions discovered by ATAC-seq was consistent with previous reports^4^. Finally, the correlation of peak signals both within and between fresh and biobanked samples was high across the large range of starting material (Fig. 1h), indicating that ATAC-seq on biobanked specimens accurately recapitulated that of fresh samples. For example, the major histocompatibility complex class II locus (MHC-II) is actively transcribed in human B cells. The *HLA-DRB1* and *HLA-DQA1* promoters were identified as accessible, as well as intergenic regions representing the XL9 insulator element^5^ and CIITA binding sites^6^ in a region classified as a super enhancer^7^ (Fig. 1i). These data show that chromatin accessibility patterns were preserved during biobanking.

**Figure 2:**
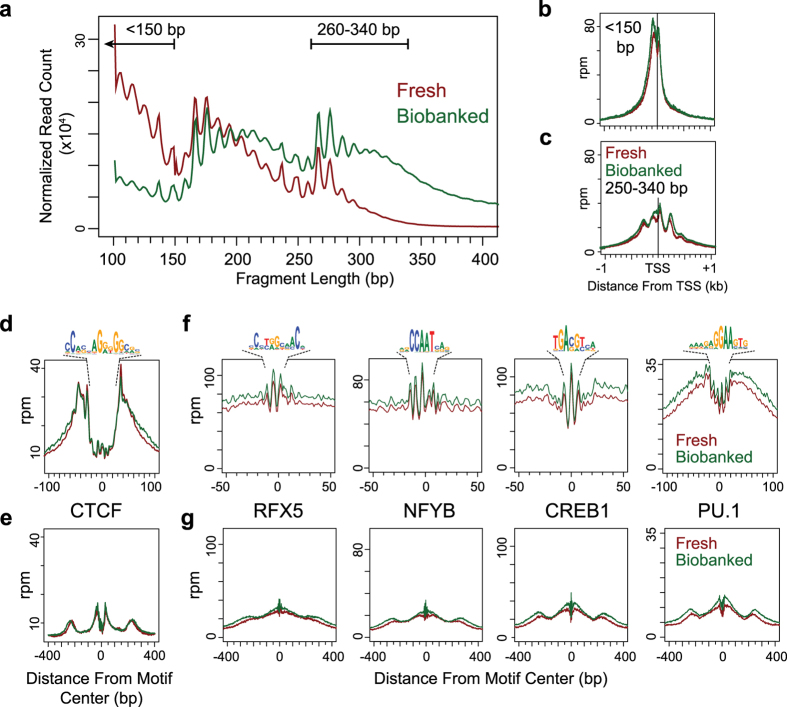
Biobanking preserves protein-DNA interaction structure. (**a**) Histogram of the distribution of fragment lengths in reads from all fresh or biobanked samples. The enriched regions of sub-nucleosomal (<150 bp) and di-nucleosomal (260–340) are indicated. Histograms of fresh and biobanked reads separated by fragment lengths of (**b**) <150 bp and **(c**) 260–340 bp at all hg19 RefSeq transcription start sites (TSS). The vertical bar indicates the position of the TSS. Histograms of fresh and biobanked reads were separated by fragment length of (**d**) <150 bp and (**e**) 260–340 bp at 56,208 CTCF motifs. The CTCF motif used for the analysis is shown above the footprint. (**f**) Histogram comparing fragments corresponding to sub-nucleosomal lengths from fresh and biobanked samples at 11,318 RFX5, 18,094 NYFB, 12,115 CREB1, and 56,420 PU.1 motifs. The motif used for each analysis is indicated. (**g**) Histogram comparing fragments corresponding to di-nucleosomal reads from fresh and biobanked samples at the transcription factor motif locations described in D.

During ATAC-seq tagmentation, distinct periodic patterns of chromatin fragmentation are observed as nucleosomes and DNA-binding proteins protect DNA from transposition events^2^. Although the distribution was distinct, the pattern of sequencing read fragment sizes was similar for both fresh and biobanked samples (Fig. 2a). Sequencing reads representing intra-nucleosomal (<150 bp) and di-nucleosomal (260–340 bp) fragments were separated and analyzed for their unique distribution pattern at genomic features. The distribution of intra-nucleosomal reads at all human RefSeq transcription start sites (TSS) showed a single peak of enrichment at the nucleosome free region (Fig. 2b). Conversely, di-nucleosomal reads displayed a periodicity surrounding the TSS, identifying the position of the upstream and downstream positioned nucleosomes (Fig. 2c), and indicating that the biobanking process had maintained TSS chromatin structure.

The footprint of mammalian transcription factors were plotted to determine if biobanking affected the ability to resolve the accessibility patterns of DNA-binding proteins. The pattern of intra-nucleosomal and di-nucleosomal reads was computed surrounding the positions of CCCTC binding factor (CTCF) binding motifs calculated from ENCODE data profiling the GM12878 lymphoblastoid cell line^8^. Intra-nucleosomal reads displayed enrichment that peaked at the motif boundaries, identifying the protected footprint where CTCF contacts DNA (Fig. 2d). In contrast, di-nucleosomal reads weakly showed the protected footprint and further identified two additional enriched regions 200 bp surrounding the motif (Fig. 2e). These patterns are similar to the locations of positioned nucleosomes surrounding CTCF binding sites^9^. Additionally, similar transcription factor accessibility footprint patterns were observed at the sequence motifs for other important B cell factors: RFX5, NFYB, CREB1, and PU.1 (Fig. 2f,g). Minimal differences in overall accessibility were observed between fresh and biobanked samples, but this did not influence the ability to observe discrete footprints. Importantly, the distribution of intra-nucleosomal and di-nucleosomal reads surrounding the TSS and transcription factor binding sites were identical in biobanked and fresh samples, indicating biobanking had no global effect on protein-DNA interactions.

### Naïve SLE B cells exhibit a unique chromatin architecture

**Figure 3:**
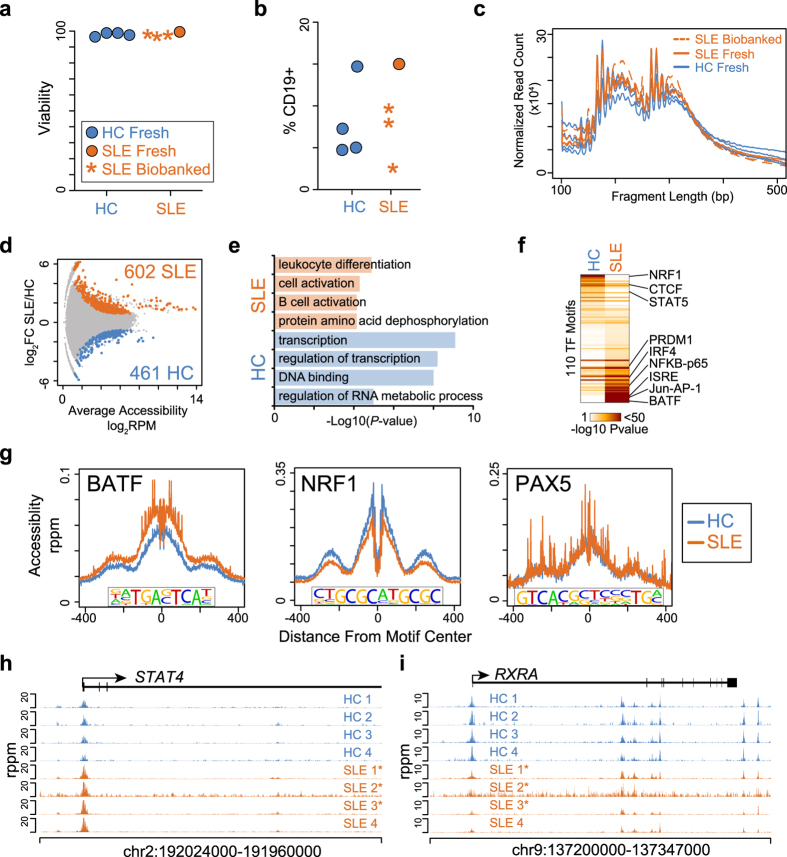
SLE B cells display an altered chromatin accessibility profile. (**a**) Summary of the percentage of viable cells for HC and SLE samples. *P*-value = 0.98. **(b**) Dot plot of the percentage of CD19^+^ naïve B cells for HC and SLE samples. *P*-value = 0.81. (**c**) Histogram of the paired-end fragment lengths of HC and SLE samples. (**d**) Scatter plot of the average accessibility at each peak in HC and SLE versus the log fold change (logFC) in accessibility. Accessible loci that are significantly differentially accessible (FDR <0.05) are highlighted in blue (HC) or orange (SLE) with the number of loci indicated. (**e**) Bar plot of GO Biological Processes enriched in SLE or HC accessible loci. **(f**) Heatmap showing the significance of 110 transcription factor motifs enriched in HC and SLE accessible loci. Motifs are sorted from the most enriched in HC to the most enriched in SLE. The locations of select motifs are highlighted. (**g**) Histogram of the accessibility at 800 bp surrounding BATF, NRF1, and PAX5 motifs identified in all accessible loci in HC and SLE. The motif identified is indicated below each histogram. Data are normalized to reads per peak per million (rppm) as described by equation 3 in Methods. Genome plot depicting the ATAC-seq profiles for HC and SLE samples at the *STAT4* (**h**) and *RXRA* (**i**) genomic loci. The positions of each gene, direction of transcription, and exon locations are indicated. *Indicates biobanked SLE samples.

SLE is characterized by increases in autoreactive B cell subsets^10^,^11^,^12^,^13^. Genetic predispositions have been identified but there is a strong implication for an epigenetic component that contributes to disease etiology^14^,^15^. Interestingly, many disease susceptibility polymorphisms, including causal ones, occur in B cell signaling pathways^16^,^17^ and frequently map to non-coding regulatory regions^18^. Recent data revealed that naïve B cells form an underappreciated component of active disease flares^11^, suggesting B cells harbor pathogenic alterations at an early stage. Therefore, it was hypothesized that an altered epigenetic program was present in naïve SLE B cells. To test this hypothesis, the ATAC-seq assay was applied to samples isolated from a biorepository of SLE patients undergoing disease flares. Three SLE samples biobanked for two years were processed in combination with one freshly obtained SLE sample. As a comparison, four healthy control (HC) patients were recruited as controls. No difference was observed in the cellular viability post-thawing of the biobanked samples compared to the fresh samples (Fig. 3a) or in the frequency of naïve CD19^+^ B cells (Fig. 3b). Naïve CD19^+^ naïve B cells were FACS isolated (Supplementary Fig. S1C) and the accessible chromatin landscape determined by ATAC-seq for each sample. All samples were highly similar with respect to the fragment size distribution of sequencing reads and the number of peaks identified (Fig. 3c).

Differentially accessible regions between SLE and HC were identified and 602 loci demonstrated significant increases in accessibility in SLE B cells while 461 loci were more accessible in HC B cells (Fig. 3d). Differentially accessible loci mapped to 988 distinct genes, including 66 genes that contained more than one differential region. Of these genes, 98% (65/66) displayed concordant changes in accessibility, suggesting coordinated changes in accessibility of potential *cis*-regulatory elements associated with disease. To define the function of SLE or HC specific accessibility changes, differential loci were annotated to the nearest gene and ontology analysis performed to identify enriched biological processes. Increases in accessibility in SLE B cells were associated with leukocyte differentiation, cellular activation, and B cell activation while HC accessible loci were enriched for processes associated with transcriptional regulation (Fig. 3e). To gain insight into the potential signaling networks programming accessibility changes, the transcription factor motifs enriched in the SLE and HC specific accessible regions were determined. Loci with increased accessibility in HC contained motifs for NRF1, CTCF and STAT5 (Fig. 3f). Contrastingly, SLE specific accessible loci displayed enrichment for transcription factors involved in B cell activation such as NFKB, AP-1, and BATF, as well as B cell differentiation factors IRF4 and PRDM1. The enrichment of these motifs in differentially accessible loci suggests that the binding of NRF1 and BATF impact local chromatin accessibility in HC and SLE, respectively. Indeed, compared to the naïve HC B cells, those from SLE patients displayed increased accessibility in the 200 bp surrounding BATF motifs present at all accessible loci within the genome (Fig. 3g). Conversely, HC B cells demonstrated increased accessibility at NRF1 motifs. No difference in accessibility was observed for a control motif, PAX5, which was not enriched in SLE or HC. These data therefore identified an activation signature in SLE B cells that is manifested in changes in chromatin accessibility surrounding specific transcription factor binding motifs.

Examples of differentially accessible loci include the *STAT4* promoter, which demonstrated higher accessibility in SLE B cells (Fig. 3h). Polymorphisms in *STAT4* are highly associated with autoantibody production^19^ and changes in the promoter accessibility of *STAT4* could result from higher IFN-alpha signaling in SLE patients^20^ or suggests that SLE B cells are epigenetically predisposed for activation of the *STAT4* pathway. HC specific accessible loci were primarily associated with genes involved in transcriptional regulation. Among the transcriptional regulators with increased accessibility in HC B cells was *RXRA* (Fig. 3i). Mice deficient in *RXRA* display increased antibodies to nuclear antigens^21^. Thus, disease-specific changes identified in the accessible chromatin landscape indicate that SLE B cells are epigenetically distinct from HC.

## Discussion

Biobanking is routinely used to store clinical samples for future experiments. For long-term studies, biobanking offers significant experimental advantages in that samples can be stored and processed in bulk, thereby reducing technical batch effects due to sample preparation. Additionally, preexisting biorepositories provide access to a vast and diverse number of specimens, thereby avoiding lengthy sample collection studies and allowing selection of specimens based on outcome data. Rigid biobanking practices are important for long-term sample preservation at the cellular phenotypic and molecular level. Metrics that measure both cellular viability and complexity are important criteria for evaluating biobanking protocols. The data presented herein suggest that measuring chromatin accessibility may be an important molecular metric for determining biobanking success.

Here the framework for applying the ATAC-seq assay to biobanked specimens is presented and was applied to a repository of PBMCs biobanked from SLE patients undergoing disease flares. To gain insight into the epigenetic programming of SLE, ATAC-seq was performed on CD19^+^ naïve B cells from SLE and HC subjects. A unique pattern was observed that indicated increases in genomic accessibility occur both surrounding genes involved in B cell activation and the transcription factor binding sites that regulate B cell activation and differentiation. The transcription factor BATF in particular has an emerging role in B cell activation and function^22^, including direct transcriptional activation of IgM and AID^23^,^24^. Additionally, BATF motifs were previously discovered to be overrepresented in the promoters of autoimmunity susceptibility genes^25^. These data, along with the presence of increased accessibility surrounding BATF motifs in SLE, suggests a previously unknown role for BATF in the etiology of SLE B cells. Currently it is not known if the accessibility signature is an intrinsic feature of SLE B cells or due to external environmental stimuli that results in the activation of signaling networks that drive changes in accessibility. Nevertheless, the finding that alterations in the epigenome converge with genetic data^17^ further pinpoint B cell activation as a key dysregulated process in SLE.

The data presented here demonstrate the feasibility of determining the accessible chromatin landscape from biobanked samples. Mechanistically, ATAC-seq facilitates the identification of *cis*-regulatory elements. In addition to the network analyses presented here, ATAC-seq has the potential to impact traditional GWAS studies that seek to relate non-coding, intergenic disease associations to regulatory elements. Therefore, chromatin accessibility profiling is a powerful addition to the toolbox of assays that can be applied to biobanked specimens.

## Methods

### Biobanking

#### PBMC isolation

Samples were obtained with informed consent in accordance with protocols approved by the Emory University School of Medicine Institutional Review Board. SLE donors fulfilled >4 revised ACR criteria for the classification of SLE^26^. PBMCs were isolated from healthy control (HC) or SLE donors by centrifugation at 1,500× G for 25 min at room temperature (RT) using cell preparation tubes (CPT) containing sodium heparin and Ficoll-Hypaque solution. The plasma layer was removed from CPTs, and PBMCs were transferred into a 50 ml conical tube and topped off to a final volume of 50 ml with PBS (Cellgro). PBMCs were pelleted by centrifugation at 1,300 RPM at RT for 10 min, and PBS was aspirated off of cell pellet. PBMCs were resuspended in 50 mL of PBS and spun at 1,300 RPM at RT for 10 min for a total of 3 washes.

#### Biobanking of total PBMCs

A biobanking protocol was developed that allowed storage and distribution of samples for the Autoimmunity Centers of Excellence program. Following PBMC isolation, samples to be frozen were slowly resuspended in 1 ml 4 °C freezing medium (heat-inactivated, filtered FBS containing 10% DMSO) at a concentration of 10 million cells/ml, placed into a RT slow-freeze container, moved to −80 °C overnight, and then placed in liquid nitrogen for long-term storage.

#### Thawing of total PBMCs

Frozen total PBMCs were removed from liquid nitrogen and placed into a 37 °C water bath until thawed (less than 2 minutes). Thawed PBMCs were transferred to a 15 ml conical tube and diluted with PBS drop-wise to 10 ml. The 15 ml conical tube was inverted to mix and spun at 1,300 RPM for 10 min at RT. Freezing media and PBS were aspirated off of the cell pellet. Cells were resuspended in 10 ml of PBS and spun at 1,300 RPM for 10 min at RT. PBMCs were then subjected to FACS.

### PBMC staining and FACS sorting of CD19^+^ B cells

Freshly isolated or thawed total PBMCs were pulsed with 20 nM of MitoTracker Green (Invitrogen, Inc.) in pre-warmed complete media (RPMI 1640 supplemented with 10% FBS and 1% L-glutamine) at 37 °C for 30 min. Cells were pelleted at 1,300 RPM for 10 min at RT, resuspended, and chased with 10 ml of pre-warmed complete media for 30 min at 37 °C. Cells were again spun at 1,300 RPM for 10 min, resuspended at 10^7^ cells per 100 μl of PBS containing 0.5% BSA, 5% normal mouse serum, and 5% normal rat serum, and stained for flow cytometry with the following fluorochrome conjugated mouse anti-human monoclonal antibodies: anti-CD3, anti-CD24 (Invitrogen, Inc.), anti-CD19, anti-IgD, anti-CD27, anti-CD38 (BD Biosciences, Inc.). Analysis was performed using a BD LSRII. Sorting was performed on a FACS Aria II. Prior to each sort the FACS AriaII was calibrated with fluorescent beads to achieve a >99% sort purity.

### ATAC-seq

ATAC-seq was performed as described previously^6^ with adaptations^2^,^27^. HC and SLE CD19^+^ naïve B cells were sorted into FACS buffer (PBS containing 10% FBS) and cells centrifuged at 500× g at 4 °C for 10 min. Cells were resuspended in 50 μl of Nuclei Lysis buffer (10 mM Tris-HCl [pH 7.4], 10 mM NaCl, 3 mM MgCl_2_, 0.1% IGEPAL CA-630, molecular grade H_2_O, filter sterilized) and immediately centrifuged at 500× g at 4 °C for 30 min. The supernatant was removed and nuclei resuspended in 25 μl of Tagmentation Reaction mix (2X TD buffer, 1 μl Tagmentation enzyme, molecular grade H_2_O, (Illumina, Inc.)). Tagmentation reaction was incubated for 60 min at 37 °C. The tagmentation reaction was stopped with 23 μl of Tagmentation Clean-up buffer (326 mM NaCl, 109 mM EDTA, 0.63% SDS) and 2 μl of 10 mg/ml Proteinase-K and incubated for 30 min at 40 °C. DNA was isolated following a negative SPRI-size selection of 0.3× followed by a positive SPRI-selection of 1.2×. Tagmented DNA was eluted in 28 μl of Tris-HCl (pH 8.0).

PCR amplification was performed by combining 28 μl of tagmented DNA with 5 μl each of i5 and i7 dual indexing primers (Nextera Indexing Kit, Illumina, Inc.), 10 μl of 5× KAPA HiFi GC Buffer with MgCl_2_ (KAPA Biosystems), 1 μl of 10 mM dNTPs, and 1 μl of KAPA HiFi HotStart Polymerase (KAPA Biosystems). An initial amplification was performed using the following cycle conditions: 1 cycle of 72 °C for 3 min, 5 cycles of 98 °C for 10 sec, 63 °C for 30 sec, and 72 °C for 30 sec. To estimate the number of PCR cycles required, 2 μl of each reaction was diluted 1:100 with 0.05% Tween-20 and quantitated using the KAPA Library Quantification qPCR Kit (KAPA Biosystems) according to the manufacturer’s protocol. The number of additional PCR cycles needed was determined by equation (1):


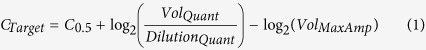


where C_Target_ is the target number of PCR cycles; C_0.5_ is the cycle number at half maximum fluorescence intensity; Dilution_Quant_ is the dilution of material added to quant; Vol_Quant_ is the volume of the library added to quant PCR number of PCR cycles; and Vol_MaxAmp_ is the maximum volume of the undiluted library to be added to the amplification PCR.

The total number of amplification cycles was normalized between samples by setting C_Target_ to the maximum C_Target_ value and calculating the volume of each sample, Vol_Amp_, to add to the reaction using equation (2):


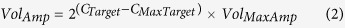


where C_MaxTarget_ is the maximum C_Target_ value of all samples. PCR amplification was completed using ‘C_Target_’ as calculated above: C_Target_ cycles of 98 °C for 10 sec, 63 °C for 30 sec, 72 °C 30 sec, and 1 cycle of 72 °C for 60 sec.

To remove primers and high molecular weight DNA following PCR amplification, a dual SPRI-size selection was performed with a 0.2× initial selection and a 0.8× final isolation. Amplified library was eluted in 25 μl Tris-HCl (pH 8.0) and quality checked on a Bioanalyzer and qPCR quantitated using the KAPA Library Quantification qPCR Kit (KAPA Biosystems). Libraries were pooled at equimolar ratios and sequenced on a HiSeq2500 using 50 bp paired-end Illumina chemistry.

### ATAC-seq Data Processing

All ATAC-seq data has been deposited in the NCBI GEO database under accession GSE71338. Raw fastq reads were checked for nucleotide distribution and read quality using the FASTX-toolkit and mapped the hg19 version of the human genome using Bowtie^28^ and the default settings. Only uniquely mapped and non-redundant reads were used for downstream analyses. Peak calling was performed with HOMER software^29^ and the ‘-style dnase’ setting. All peaks between fresh and biobanked samples are listed in Supplementary Table S1. Reads per peak per million (rppm) normalization on HC and SLE samples was performed by equation (3):


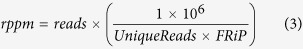


### Differential Accessibility Analysis

Significantly different accessible regions between HC and SLE B cells were determined by computing the overlap of all HC and SLE peaks using the HOMER ‘mergePeaks’ function. The raw, non-normalized reads from each sample were annotated for each peak using the ‘annotatePeaks.pl’ script with the following options ‘-size given –noadj’. The resulting composite peak file was used as input for edgeR^30^ using the ‘getDiffExpression.pl’ HOMER script with the following options ‘-peaks HC HC HC HC SLE SLE SLE SLE’. Peaks with an FDR <0.05 were considered significantly differentially accessible between HC and SLE B cells. All significant peaks are listed in Supplementary Table S2. Differentially accessible peaks were annotated to the nearest gene using the ‘annotatePeaks.pl’ HOMER script and motif enrichment calculated using the ‘findMotifsGenome.pl’ script. Ontology analysis of genes with increases and decreases in accessibility between HC and SLE was performed using DAVID^31^,^32^.

### Fragment Length Analysis

Bam files were parsed and the fragment length analyzed using the GenomicAlignments^33^ R/Bioconductor package. Qnames corresponding to reads with a fragment length of <150 bp or between 250 and 340 bp were extracted. The bam files were converted to sam files using the samtools package^34^,^35^ and parsed for reads with desired fragment lengths based on extracted Qnames with custom python scripts. Fragment-length specific sam files were used as input for HOMER to generate tag directories using the ‘makeTagDirectory –format sam’ script. All custom scripts are available upon request.

### Motif Histograms

The ‘wgEncodeRegTfbsClusteredV3.bed.gz’ file^8^,^36^,^37^ was downloaded from the UCSC Genome Browser and binding sites for CTCF, PU.1, RFX5, NFYB, CREB1 extracted using custom Perl scripts. Motif positions in peaks were identified using FIMO^38^ and position weight matrices for each transcription factor acquired from the JASPAR database^39^. The highest scoring motif in each peak was chosen for further analyses. Motif coordinates were used as input for the HOMER findPeaks.pl script using the ‘-size 1000 –fragLength 1 –norm 1e6 –hist’ options.

## Additional Information

**Accession codes**: NCBI Gene Expression Omnibus: sequencing data are available under the accession number GSE71338. 

**How to cite this article**: Scharer, C. D. *et al*. ATAC-seq on biobanked specimens defines a unique chromatin accessibility structure in naïve SLE B cells. *Sci. Rep.* **6**, 27030; doi: 10.1038/srep27030 (2016).

## Supplementary Material

Supplementary Information

Supplementary Table S1

Supplementary Table S2
