# Supplementary material for: ATAC-seq on biobanked specimens defines a unique chromatin accessibility structure in naïve SLE B cells
Source: Sci Rep. 2016 Jun 1;6:27030. doi: 10.1038/srep27030 (PMC4888756; doi:10.1038/srep27030)
Supplement: Supplementary Information [file srep27030-s1.pdf]

ATAC-seq on biobanked specimens defines a unique chromatin accessibility structure in naïve SLE B cells

Christopher D. Scharer[1,+], Emily L. Blalock[2,+] , Benjamin G. Barwick[1], Robert R. Haines[1], Chungwen Wei[2], Ignacio Sanz[2], and Jeremy M. Boss[1,*]

[1]Department of Microbiology and Immunology, Emory University School of Medicine, Atlanta, GA

[2]Department of Rheumatology, Emory University School of Medicine, Atlanta, GA

*jmboss@emory.edu

[+]These authors contributed equally to this work

Supplementary Table S1. ATAC-seq peaks identified in fresh and biobanked samples.

Supplementary Table S2. ATAC-seq peaks significantly differentially accessible between HC and SLE subjects.

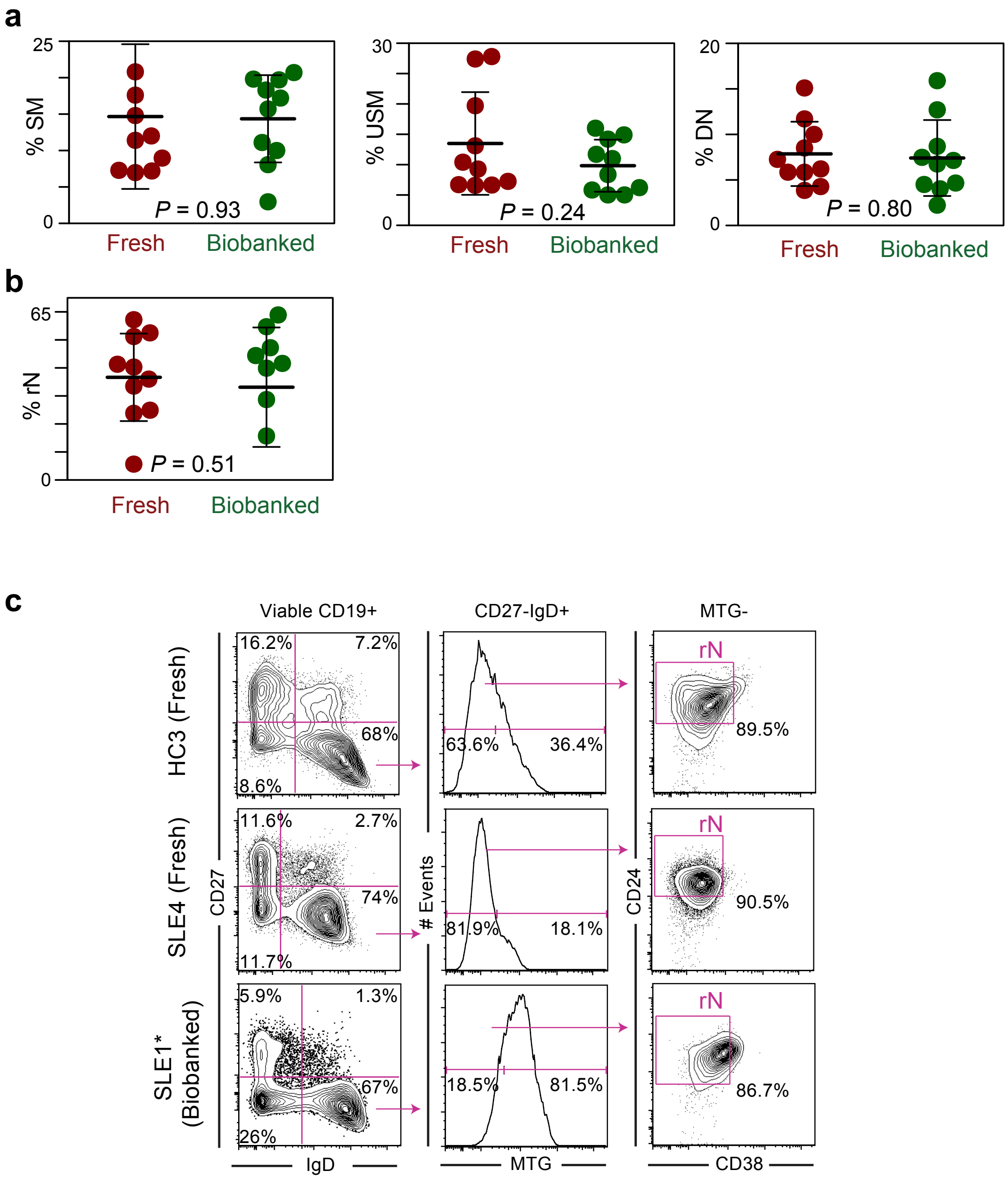

Supplementary Figure 1. Flow cytometry data of B cell populations. **(A)** Dot plots displaying the percentage of CD19+ B cell populations that are Switched Memory (SM), Unswitched Memory (USM), and Double Negative (DN) cells. Data is from 10 fresh and 10 biobanked samples. Statistical significance was tested by Student's *t*-test. **(B)** Dot plot representing the percentage of resting naïve (rN) B cells in 10 fresh and 10 biobanked samples. Statistical significance

was tested by Student's *t*-test. **(C)** Flow cytometry plots showing the gating strategies to isolate resting naïve B cells (rN) from HC and SLE subjects. The percentage of each population is indicated. A representative fresh HC (HC3) and biobanked SLE (SLE1*) are shown along with the fresh SLE sample (SLE4).
